# Patient Plan Customization in Hypofractionated CyberKnife Radiosurgery for Extensive Brain Metastases Within a Resource-Constrained Low-Middle-Income Country

**DOI:** 10.7759/cureus.92163

**Published:** 2025-09-12

**Authors:** Ayesha Ahmad, Haseeb Mehmood Qadri, Noraiz Rasool, Asif Bashir

**Affiliations:** 1 Radiation Oncology, Ghurki Trust Teaching Hospital, Lahore, PAK; 2 General Surgery, Lahore General Hospital, Lahore, PAK; 3 Neurological Surgery, Punjab Institute of Neurosciences, Lahore, PAK; 4 Medical Physics, Ghurki Trust Teaching Hospital, Lahore, PAK

**Keywords:** 60 mets, brain mets, breast cancer, breast secondaries, cyberknife, metastatic brain disease, pakistan, radiosurgery

## Abstract

Brain metastasis (BM) carries a dismal prognosis. In the setting of advanced breast cancer, BM has a formidable outcome, yet radiosurgery has played a pivotal role in palliating patients. Here, we present the case of a 62-year-old hypertensive female with biopsy-proven, triple-negative, invasive ductal carcinoma of the left breast (stage IV disease). Whole-brain radiotherapy (WBRT) failed to achieve radiological and clinical response for 60 intracranial metastatic space-occupying lesions. Her presenting complaint of intractable headache was accompanied by a normal neurological examination. A customized plan for stereotactic radiosurgery via CyberKnife S7 was utilized with a 2,200 cGy dose and 68% isocurve over five fractions on alternate days. Redo WBRT was not performed owing to the patient’s comorbidities, age, and risk of cognitive deficits. Radiological remission of 81% and 88% with no neurological deficits was achieved at the fourth and eighth months of follow-up. To our knowledge, this is the first case of 60 secondaries in the human brain from a breast primary successfully managed with CyberKnife stereotactic radiosurgery, achieving remarkable radiological regression while preserving clinical function. A customized plan considering the patient’s factors and risk versus benefit assessment is advisable in all cases of metastatic brain disease.

## Introduction

Malignant neoplasms of the lung, breast, colon, and melanomas are known to metastasize to the brain most commonly [1]. While lung cancer is the most common source of intracranial secondaries in males, breast cancer is the equivocal counterpart in the female population [1]. Stelzer has documented an incidence of 10/100,000 population in various population-based analyses conducted in various parts of the world [2]. The differential behavior of primary sources of cancer based on their histopathology and molecular characteristics, levels of permeability of the blood-brain barrier for chemotherapeutic drugs, and the actual micro-environment in the brain developed by the neoplasm for itself pose grave challenges in treating brain metastases (BMs) [1,2].

The ability of breast cancer (BrCA) cells to undergo epithelial-to-mesenchymal transition at the primary site and then reverse mesenchymal-to-epithelial transition at the secondary site (central nervous system) demonstrates their tremendous power to proliferate and penetrate the brain [3]. According to the Epidemiological Strategy and Medical Economics, the risk of developing BMs in patients with advanced breast cancer is as high as 25% [4]. Silmane et al. demonstrated that estrogen receptor (ER) negativity, high histological grade, and age less than 40 years are significant risk factors for BMs in BrCA [5]. Conventionally, surgical resection and radiotherapy remain the preferred options to treat BMs; however, the effects of chemotherapy and targeted therapy are being investigated in the treatment of BMs secondary to BrCA [2,3]. Furthermore, whole-brain radiation therapy (WBRT) and stereotactic treatment in the form of CyberKnife and Gamma Knife are the main forms of radiosurgery being offered [1,3].

From a resource-limited setting, we present the successfully managed case of a female with 60 BMs secondary to BrCA treated by the innovative technique of non-invasive, image-guided, robotic arm-mounted, extremely precise stereotactic radiosurgery (SRS) treatment.

## Case presentation

A 62-year-old female, a known case of hypertension, was diagnosed with triple-negative invasive ductal carcinoma grade III in March 2023. She was a restaurant manager by profession with a Karnofsky Performance Scale score (KPS) of 100. She completed neo-adjuvant chemotherapy with four cycles of Adriamycin and cyclophosphamide, followed by four cycles of carboplatin and paclitaxel. Following adequate local response, she underwent a modified radical mastectomy in 2023 and adjuvant local chest wall radiotherapy of 40 Gy in 15 fractions via a linear accelerator (LINAC).

She complained of a headache for three months; however, no neurological deficits were noticeable upon examination. Further workup was suggestive of stage IV BrCA with metastases in the brain. Neuro-navigation-guided MRI of the brain was suggestive of multiple variable-sized cortical, subcortical, and intra-axial enhancing brain lesions in bilateral cerebral hemispheres and posterior fossa. The largest two lesions in the right frontal lobe and right cerebellum measured 12.5 × 14 × 9.4 mm and 13 × 17 × 12 mm, respectively. A neuro-navigation-guided biopsy of the frontal lesion was performed to confirm receptor status and was still suggestive of triple-negative metastatic invasive ductal carcinoma.

After a multidisciplinary team meeting, she was referred for conventional radiotherapy, receiving WBRT in May 2024, amounting to 20 Gy in five fractions, while oral capecitabine 2,500 mg/m^2^ was continued. The persistent presenting complaint of intractable headache with no neurological deficits and lack of radiological disease regression in response to WBRT were the reasons for her referral to the Department of CyberKnife, Gurkhi Trust Teaching Hospital, Lahore, in June 2024.

A team meeting was conducted at our center with a neuroradiologist mapping 60 lesions of BMs at various locations (Video 1). The patient was classified as Eastern Cooperative Oncology Group performance status scale 0 and prepared for frameless stereotactic CyberKnife radiosurgery using Accuray Precision version 33.13(2). We chose this approach considering her comorbidities, profession, and to avoid escalating neuro-cognitive dysfunction (owing to her age and prior WBRT).

**Video 1:** Contrast-enhanced MRI of the brain showing 60 intracranial metastatic deposits from the primary left breast invasive ductal carcinoma.

Details of the WBRT plan were requested from the referring center. After a precise review of the prior radiation plan, a CT simulation of the brain was performed. Fusion of her CT brain with MRI slices of 1 mm thickness was done precisely. The gross tumor volume (GTV) and organs at risk (OARs) were contoured. She was given 2,200 cGy radiation with a 68% isocurve, implying 440 cGy/fraction for five fractions under thermoplastic masks on alternate days instead of the daily fraction regimen. Dosimetric evaluation revealed a dose gradient index of 1.87, suggesting an adequate peripheral dose fall-off. The Paddick conformity index was 1.10, indicating reasonable conformity between the planning target volume and the prescribed isodose distribution. A fixed collimator with sizes 5 mm, 7.5 mm, and 10 mm was used under high dose resolution. The GTC was 11.83 cm^3^ targeted within an estimated time of 123 minutes, with 467 non-zero beams and 334 imaging beams. Doses to the optic pathway, brainstem, and normal brain parenchyma were within the permissible range (Table 1).

**Table 1:** Organs at risk for various organs exposed to CyberKnife with dosage and volume exposed.

Organs at risk	Permissible dose	Volume exposed
Brainstem	18.25 Gy	0.03 cm³
Whole brain	21.78 Gy	10 cm³
Optic chiasm	7.57 Gy	0.03 cm³
Right optic nerve	1.84 Gy	0.03 cm³
Left optic nerve	8.74	0.03 cm³
Eyes (both)	No radiation	Blocked

Vitals were monitored daily before and after her treatment. The patient was stable at the completion of fractions. Concurrently, steroids were given during the protocol treatment (Table 2). She was discharged on a tapering dose of steroids and analgesics for headache on the 12th post-admission day in good condition. Psychological support and counseling sessions were offered to the patient during management.

**Table 2:** Steroid and proton pump inhibitor regimen administered during and after radiosurgery.

Medication	Dosage and frequency	Duration	Notes
Proton pump inhibitor	Pantoprazole 40 mg once daily, orally	Throughout the regimen	Administered in the morning on an empty stomach
Dexamethasone	16 mg/day divided as 4 mg every 6 hours (Q6H), orally	2 weeks	Given on alternate days of radiosurgery
Dexamethasone	12 mg/day divided as 4 mg every 8 hours (Q8H), orally	4 days	First taper after radiosurgery completion
Dexamethasone	8 mg/day divided as 4 mg every 12 hours (Q12H), orally	4 days	Second taper
Dexamethasone	4 mg/day divided as 2 mg every 12 hours (Q12H), orally	4 days	Third taper
Dexamethasone	2 mg once daily (OD), orally	4 days	Maintenance dose before the final taper
Dexamethasone	0.5 mg once daily (OD), orally	4 days	Final taper before stopping

**Figure 1:**
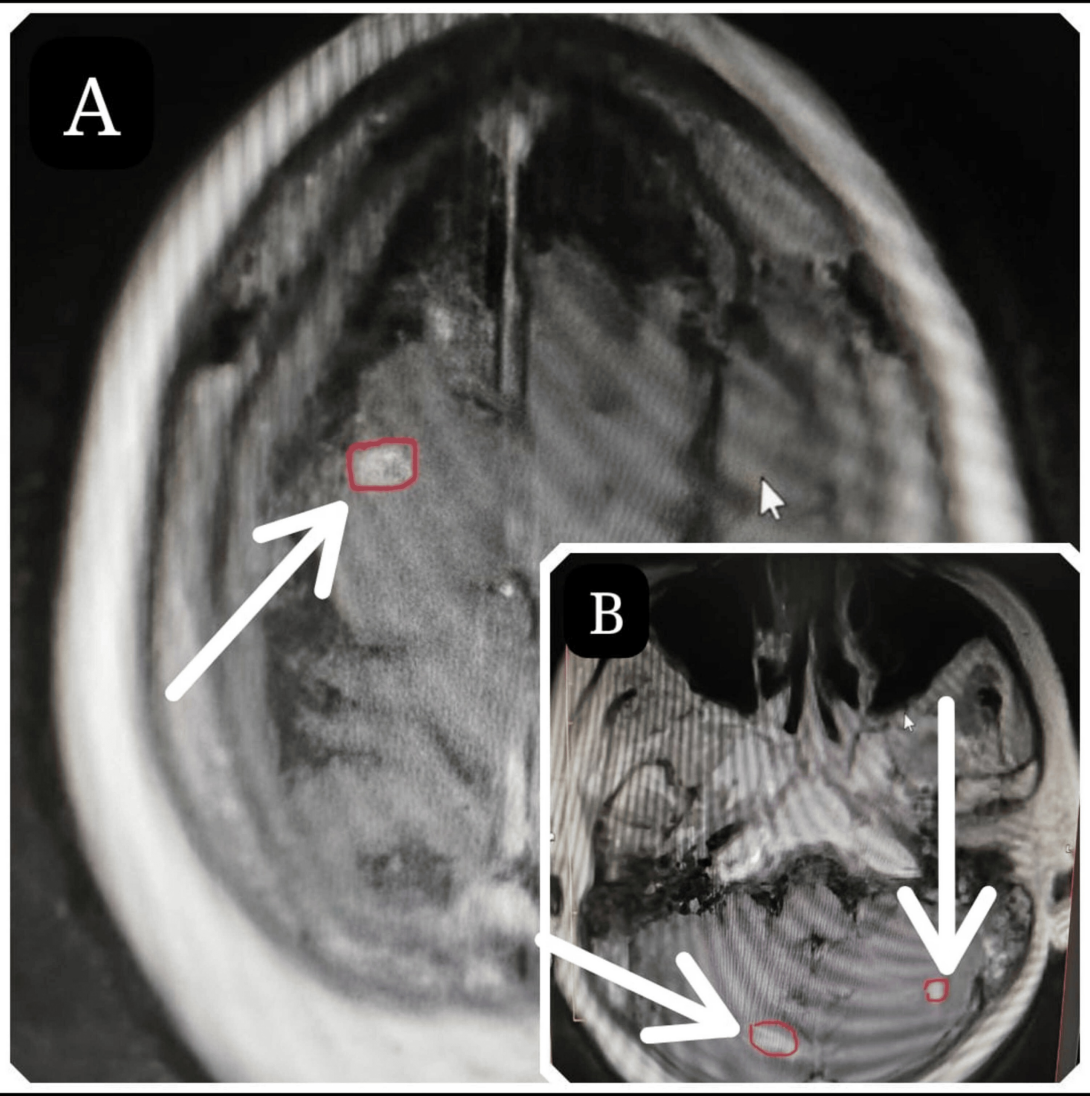
Contrast-enhanced MRI of the brain at the fourth month of follow-up showing right frontal (A) and bilateral cerebellar (B) metastatic deposits (white arrows) from the primary left breast invasive ductal carcinoma.

At fourth-month follow-up, the right frontal lobe and right cerebellar lesions regressed to 8.4 × 8.4 × 8.5 mm and 5.7 × 15.8 × 5.3 mm on contrast-enhanced MRI, respectively, implying a remarkable overall actuarial regression of 81% (Figure 1).

**Figure 2:**
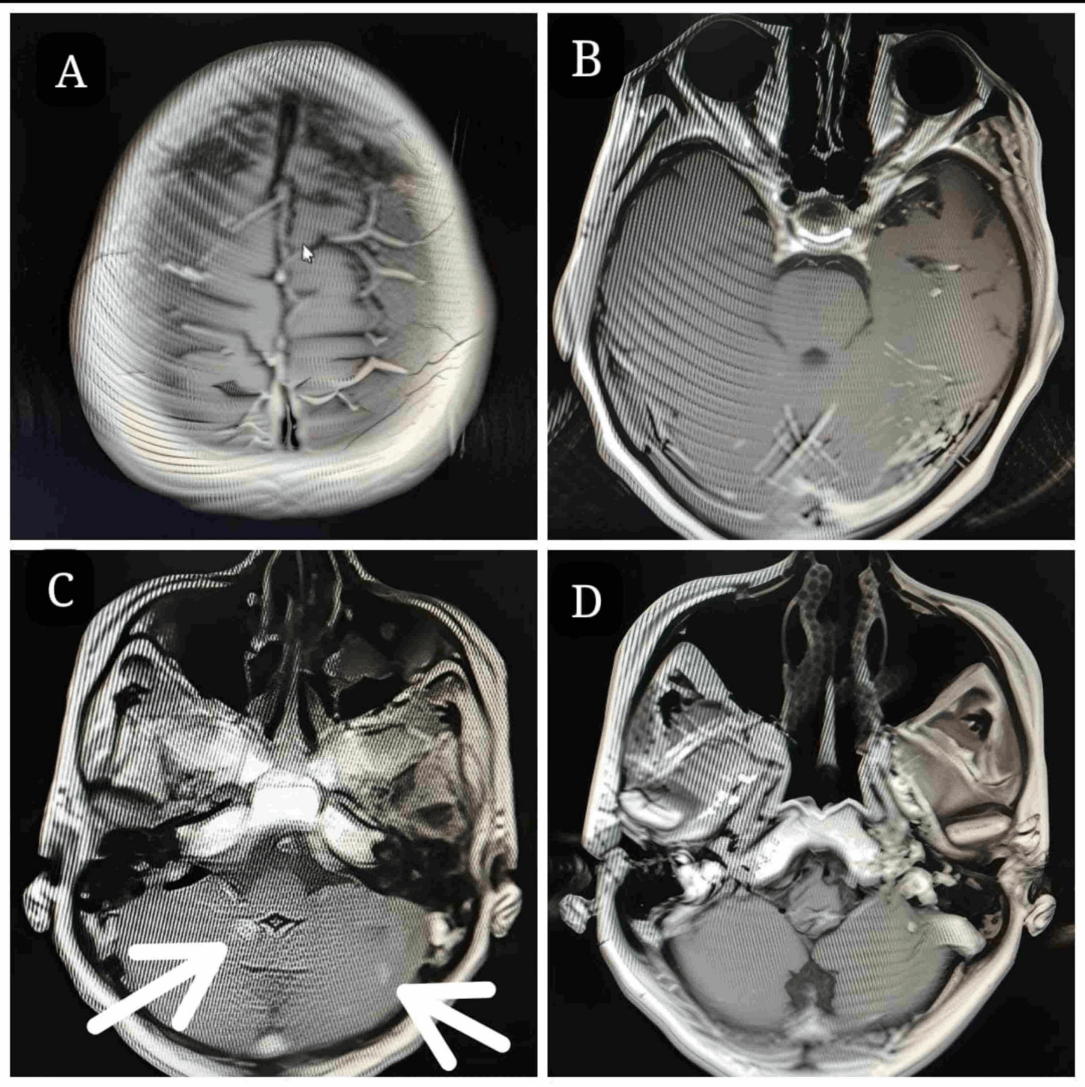
Contrast-enhanced MRI of the brain at the eighth month follow-up with almost complete regression (A, B, and D) of intracranial metastatic deposits of the primary left breast invasive ductal carcinoma and residual deposits (C). (A) Axial section with parts of frontal, parietal, and occipital lobes. (B) Axial sections of the temporal, parietal, and occipital lobes. (C) Axial section of mid-cerebellum with white arrows indicative of residual metastatic deposits. (D) Axial section of the lower cerebellum and brainstem.

On the eighth-month follow-up, contrast-enhanced MRI of the right frontal and right cerebellar lesions showed considerable regression to 4 × 6 × 2 mm and 4.6 × 4.9 × 2.1 mm, respectively, implying a remarkable overall actuarial regression of 88% (Figure 2). Most of the smaller intracranial lesions resolved completely. Due to a significant measurable reduction in target lesions, the outcome according to Response Assessment in Neuro-Oncology Brain Metastases was classified as partial response. The patient is living a healthy life with no neurological deficits.

Informed written consent for the publication of neuroimaging and patient data was obtained from the patient herself.

## Discussion

To the best of our literature search using PubMed, this is the first case of metastatic brain disease with 60 space-occupying lesions from a primary source of BrCA. The following factors can be attributed to the successful management of this case: the fact that the number of secondaries in metastatic brain disease is actually underscored by the volume of the metastatic lesions altogether, in the form of GTV. Though 60 in totality, the GTV in our study was 11.83 cm³, manageable with CyberKnife radiosurgery and preserving the normal brain parenchyma. Second, we made use of alternate-day fractions, rather than daily fractions. More so, concurrent utilization of low-dose steroids and a thermoplastic mask, critical vital monitoring pre-session and post-session radiosurgery, and comprehensive psychological support played a significant role in preventing damage to OARs and ensuring the overall mental well-being of our patient.

In a series of 34 German patients with BMs, Telentschak et al. documented a female preponderance of 61.76% irrespective of the neoplasm histopathology [6]. While a Mexican study published in 2018 with 49 patients showed a male majority with 59% cases irrespective of tumor types [7]. In our study, the patient was an elderly female. It is difficult to assess the gender predilection for BMs in general due to scanty studies on the epidemiology of cerebral metastases. However, supported by the literature, lung cancer is a leading cause of BMs in males, while breast cancer remains on top as a source of cerebral secondaries in females [1]. ER negativity, triple-negative status, grade III on histopathology, number and location of extra-cerebral metastases, and short interval of time between diagnosis of primary and metastatic disease are tightly associated risk factors for BMs in BrCA [3]. Similarly, our patient had triple-negative status, a higher histopathology grade, and a short time interval for the development of secondaries from the primary source, reinforcing the significance of risk factors.

In our case, a single-fraction radiosurgery plan was initially considered; however, the total beam-on time for such a plan was approximately 256 minutes (over four hours), making it clinically impractical. The prolonged treatment duration would have required the patient to remain immobilized in a stereotactic fixation device for an uncomfortably long period, which was not feasible. Therefore, a fractionated treatment approach was selected to ensure better patient compliance and comfort while maintaining dosimetric safety and efficacy. Although a dose of 2,500 cGy in five fractions is commonly used as per institutional standards for SRS, a reduced dose of 2,200 cGy in five fractions was selected in this case. This decision was based on the patient’s history of prior irradiation and the proximity of the target lesion to critical normal brain structures. The lowered dose was chosen to minimize the risk of toxicity, particularly to the normal brain parenchyma, as the cumulative dose needed to remain within acceptable limits for re-irradiation. The modified regimen balanced the need for effective local control while adhering to established constraints for normal tissue tolerance.

The role of WBRT in treating BMs remains controversial owing to its established association with a decline in cognition. Brown et al. conducted a randomized controlled trial comparing the effect of SRS alone versus SRS with WBRT in patients with one to three BMs. Although SRS with WBRT achieved better local and distant control than SRS alone, the WBRT group was associated with profound cognitive deficit and lower quality of life [8]. Contrastingly, our patient did not develop any cognitive deficits after WBRT, but the local control for BM was poor, and the lesions grew in number. Andrews et al. documented the results of phase III Radiation Therapy Oncology Group (RTOG) 9508, where they administered WBRT or WBRT followed by SRS boost to 333 newly diagnosed cases of one to three BMs randomly. The SRS boost was given only a week after WBRT in the latter group. Patients in the latter group had a better overall KPS score after six months of treatment [9]. Similarly, our patient received CyberKnife radiosurgery after poor WBRT response, yielding a post-CyberKnife radiosurgery KPS score of 100 until the last follow-up.

Radiosurgery platforms are mainly classified as cobalt-based, LINAC-based, and robotic. There is no consensus to date upon which type of platform is suited to which kind of BMs. Gamma Knife makes use of cone-beam CT, while CyberKnife utilizes yet more effective six-dimensional skull-based motion trackers to monitor the patient’s position. In their contemporary review published in 2022, Skourou et al. concluded that Gamma Knife and CyberKnife provide an effective response at the cost of treatment duration [10]. Optimum delivery of maximum dose to GTV was achieved in our case, with minimal radiation to the peripheral tissue due to effective patient motion monitoring. This is evident from the clinical assessment, i.e., normal neurology and radiological follow-ups of our patient. Myrehuag et al. studied the effects of hypofractionated stereotactic radiation therapy upon 220 patients with 334 BMs and found local failure of 13% and 33% at 6 months and 12 months, respectively. However, with a 22 Gy dose, we achieved local control at the fourth and eighth months of follow-up with regression of lesions in our patient [11].

## Conclusions

A total of 60 intracranial metastatic space-occupying lesions secondary to BrCA are the largest number of metastatic lesions reviewed across existing scientific literature. The cessation of the complaint of persistent headaches with normal neurological function, a post-radiosurgery KPS score of 100, and actuarial metastatic regression of 88% imply that hypofractionated CyberKnife radiosurgery is an effective robotic platform to address multiple intracranial metastases. Patient factors and resources should be thoroughly considered in resource-limited settings when WBRT fails to achieve local control.

## References

[1] Aleksandrovic E, Zhang S, Yu D (2024). From pre-clinical to translational brain metastasis research: current challenges and emerging opportunities. Clin Exp Metastasis.

[2] Stelzer KJ (2013). Epidemiology and prognosis of brain metastases. Surg Neurol Int.

[3] Bailleux C, Eberst L, Bachelot T (2021). Treatment strategies for breast cancer brain metastases. Br J Cancer.

[4] Darlix A, Louvel G, Fraisse J (2019). Impact of breast cancer molecular subtypes on the incidence, kinetics and prognosis of central nervous system metastases in a large multicentre real-life cohort. Br J Cancer.

[5] Slimane K, Andre F, Delaloge S (2004). Risk factors for brain relapse in patients with metastatic breast cancer. Ann Oncol.

[6] Telentschak S, Ruess D, Grau S (2021). Cyberknife(®) hypofractionated stereotactic radiosurgery (CK-hSRS) as salvage treatment for brain metastases. J Cancer Res Clin Oncol.

[7] de la Peña C, Guajardo JH, Gonzalez MF, González C, Cruz B (2018). CyberKnife stereotactic radiosurgery in brain metastases: a report from Latin America with literature review. Rep Pract Oncol Radiother.

[8] Brown PD, Jaeckle K, Ballman KV (2016). Effect of radiosurgery alone vs radiosurgery with whole brain radiation therapy on cognitive function in patients with 1 to 3 brain metastases: a randomized clinical trial. JAMA.

[9] Andrews DW, Scott CB, Sperduto PW (2004). Whole brain radiation therapy with or without stereotactic radiosurgery boost for patients with one to three brain metastases: phase III results of the RTOG 9508 randomised trial. Lancet.

[10] Skourou C, Hickey D, Rock L (2021). Treatment of multiple intracranial metastases in radiation oncology: a contemporary review of available technologies. BJR Open.

[11] Myrehaug S, Hudson J, Soliman H (2022). Hypofractionated stereotactic radiation therapy for intact brain metastases in 5 daily fractions: effect of dose on treatment response. Int J Radiat Oncol Biol Phys.

